# Depression among Adult HIV/AIDS Patients Attending ART Clinics at Aksum Town, Aksum, Ethiopia: A Cross-Sectional Study

**DOI:** 10.1155/2019/3250431

**Published:** 2019-02-03

**Authors:** Berhe Beyene Gebrezgiabher, Teklehaymanot Huluf Abraha, Etsay Hailu, Hailay Siyum, Getachew Mebrahtu, Berihu Gidey, Mebrahtu Abay, Solomon Hintsa, Teklit Angesom

**Affiliations:** ^1^Department of Epidemiology and Biostatistics, School of Public Health, College of Health Sciences, Aksum University, Aksum, Ethiopia; ^2^Department of Reproductive Health, School of Public Health, College of Health Sciences, Aksum University, Aksum, Ethiopia; ^3^Department of Psychiatric Nursing, College of Health Science, Aksum University, Aksum, Ethiopia; ^4^Department of Human Nutrition, School of Public Health, College of Health Science, Aksum University, Aksum, Ethiopia

## Abstract

**Background:**

Depression is consistently associated with increased risk of Human Immunodeficiency Virus infection and poor antiretroviral treatment adherence. Though many factors have been reported as determinant factors of depression, site-specific evidence is needed to identify factors associated with depression among adults on antiretroviral treatment.

**Methods:**

An institution based cross-sectional study was carried out from March to May 2015 among 411 adults HIV/AIDS patients on ART clinic follow-up. Participants were selected using systematic random sampling techniques. Data were collected using chart review and interviewer- administered techniques. Both bivariable and multivariable logistic regressions were used to compute the statistical test associations by SPSS version-20. Variables with p value < 0.05 were considered as statistically significant.

**Results:**

Four hundred eleven patients with a mean age ± Standard Deviation of 36.1±9.2 years and with a total response rate of 97.6% were enrolled in the study. The prevalence of depression was 14.6% (95% CI, 10.90-18.2). Factors independently associated with depression were nonadherence to ART, eating two meals per day or less, having side effect of ART medication, being in the WHO Stage II or above of HIV/AIDS, and living alone with AOR (95% CI) of 3.3 (1.436, 7.759), 2.8 (1.382, 5.794), 4.7 (1.317, 16.514), 2.8 (0.142, 0.786), and 2.4 (1.097, 5.429), respectively.

**Conclusion:**

Though the magnitude of depression was found relatively low, it was commonly observed as a mental health problem among adult patients with HIV/AIDS on ART. Programs on counseling and close follow-up of adherence to ART, drug side effects, and nutrition should be strengthened. Health facilities should link adult patients with HIV/AIDS who live alone to governmental and nongovernmental social supporter organizations.

## 1. Background

Human immune deficiency virus, HIV/AIDS, is a cause of death and disability, especially in low- and middle-income countries [1]. Currently, Sub-Saharan African is home to about 36.7 million people living with the virus, making it the most affected in the world [2]. Data from Ethiopian Federal HIV/AIDS Prevention and Control Office (EFHAPCO) indicates that there are over 718,550 people (a little over 1.18% of the population) living with HIV in Ethiopia alone [3]. The increasing access to highly effective Antiretroviral Therapy (ART) for people living with HIV (PLWHA) has delayed HIV disease progression and prolonged survival bringing into sharp focus issues of quality of life including their mental wellbeing [4].

Depression is a common mental disorder that presents with depressed mood, loss of interest or pleasure, decreased energy, feelings of guilt or low self-worth, disturbed sleep or appetite, and poor concentration [5]. It interferes with daily life and reduces the quality of life [6]. Depending on the local context, people with chronic health conditions may be placed at a significantly higher risk of experiencing mental health problems [5]. Approximately, 350 million people are currently living with depression. It is the fourth leading cause of disability worldwide, and it will become the second leading cause of disability by 2020. Its lifetime prevalence was estimated to be approximately 3 to 17 % [7].

Depression is the most common neuropsychiatric complication of HIV disease [8]. People who are infected with HIV are more likely than the general population to develop depression [6]. Mental health problems are associated with increased risk of HIV infection and AIDS and interfere with their treatment, and, conversely, some mental disorders occur as a direct result of HIV infection [9]. Depression affects a person's ability to follow treatment for HIV/AIDS [6]. It is associated with poor adherence to ART leading to immunological failure and may independently increase HIV progression [10]. Individuals with HIV/AIDS depression have been consistently associated with poor ART adherence [10, 11].

Across low-, middle-, and high-income countries, rates of depressive symptoms among adult HIV/AIDS patients on ART ranged from approximately 13% to 78%. Depression in PLWHA could be triggered by stress, difficult life events, side effects of medications, or the effects of HIV on the brain, and it might even accelerate HIV's progression to AIDS [12]. Treating depression can help to manage HIV/AIDS and improve overall health [6]. In different studies so far, sex, having comorbid TB illness, perceived HIV stigma, poor social support, HIV stage III, and poor medication adherence were found significantly associated with depression. But site-specific evidence is needed to identify factors associated with depression among adult patients with HIV/AIDS on ART. Moreover it is crucial to identify patients with depression for proper management of the disease. Thus, this research was aimed at providing data on the prevalence and factors associated with depression among adult patients with HIV/AIDS.

## 2. Methods

### 2.1. Study Design and Setting

An institution based cross-sectional study was carried out from March 20, 2015, to May 15, 2015, in Aksum town, Tigray regional state of Ethiopia. Aksum is located 1067 K/m north of Addis Ababa which is the capital city of Ethiopia. In Aksum town ART clinics, the adults ever enrolled and on ART were 2764 and 1351, respectively [13]. All adult HIV/AIDS patients (15 and above) who had at least three consecutive visits before data collection period were considered as a study population of this study.

### 2.2. Sample Size and Sampling Procedure

The sample size was calculated using a single population proportion formula considering the following assumptions: proportion of depression 45.8% [14], 5% level of significance, 95% confidence level, 5% nonresponse rate, and 5% marginal error. Accordingly, 421 adult HIV/AIDS patients on ART were included in the study. Baseline data was collected using a clinical record review. Participants were selected using systematic random sampling techniques in every other three appointees. Finally, adding the sampling interval to the preceding number, the next client was selected consecutively until the sample size is completed. Whenever the sampled client did not fulfill the inclusion criteria, immediately the next one, who was eligible, was interviewed.

### 2.3. Data Collection and Analysis

Data were collected using chart review and interviewer-administered techniques by four diploma nurses, supervised by two bachelor nurse professionals. The structured questionnaire consisted of sociodemographic, socioeconomic, nutritional, medical, and psychological variables. One-day training on the objective of the study, confidentiality of information, and interviewing techniques was given. Baseline information was extracted from ART registration and follow-up forms.

Patient Health Questionnaire (PHQ), a validated depression screening with 9-items, with score ranges from zero to 27, was used to assess depression level of the study participants [15, 16]. Initially, individuals, upon their response, were categorized into five categories: a score of 1 to 4 no depression, 5 to 9 mild depression, 10 to 14 moderate depression, 15 to 19 moderately severe depression, and 20 to 27 severe depression. In this study, a positive depression screen was defined as a PHQ-9 score greater than 9.

Adherence to ART was defined as taking one's medicine as prescribed and agreed between the patient and provider. Patients who adhere 95% or more to ART medication were considered as adherent. This means taking doses within two hours before or after the time of a doctor's advice to take it (95% or more adherence = missing ≤ 2 doses of 30 doses or ≤ 3 doses of 60 doses) [16–18].

Precoding and manual checking of the questionnaire were done by the principal investigator before data entry. Data were entered using Epi-Info and exported to SPSS version 20 for further cleaning and analysis. Binary logistic regression model was used to identify the determinant factors. The reliability coefficient, Cronbach's alpha for the PHQ-9 total score, was 0.73 which is an acceptable internal consistency. Colinearity diagnostic test was conducted to check for colinearity between independent variables and the highest colinearity; tolerance = 0.516 and VIF = 1.938 were found between side effect of ART medication and adherence to ART. Variables in the bivariable analysis having a p value < 0.2 were considered for multivariate analysis to adjust the confounders. The strength and presence of statistical association were assessed using Adjusted Odds Ratio (AOR) with 95% confidence interval (CI). Variables with a p value ≤ 0.05 in the final model were considered as statistically determinant factors of depression. Model fitness was also checked by using Hosmer–Lemeshow Goodness-of-Fit Test (p = 0.391), Deviance Goodness-of-Fit Test (p =0.942), and Pearson Goodness-of-Fit Test (p =0.217), which indicates the model fits the data well.

## 3. Results

### 3.1. Sociodemographic and Economic Related Characteristics

A total of 421 adult HIV/AIDS patients, who were on ART for at least 6 months prior to the study, were planned to be included in the study. Out of these, 411 individuals were enrolled during the data collection, with a total response rate of 97.6%. The majority, 239 (58.2%), of the study participants were males. The mean age ± (Standard Deviation) of the participants was 36.1±9.2 years. Almost half of the respondents (45.5%) were in the age of 30–39 years (Table 1).

### 3.2. Depression Level and Clinical Related Characteristics

Of the total study participants, 60 (14.6%) had depression symptoms (95% CI = 10.9% - 18.2%). Majority of the participants (76.4%) were on ART medication for more than two years. Two-thirds of the participants (66.7%) were adherent to their ART medication. Two hundred thirty-four (56.9%) of the study participants took their ART medication twice a day. Two hundred fifty-six (62.3%) of patients were living with their families (Table 2).

### 3.3. Factors Associated with Depression

During the multivariable analysis, nonadherence to ART, eating two meals per day or less, having a side effect of ART medication, being WHO HIV/AIDS clinical stage II or above, and living alone had shown significant association with depression.

Patients who were nonadherent to ART [AOR= 3.3, 95% CI (1.436, 7.759)] were 3.3 times more depressed as compared to their adherent counterpart. Study participants who eat two meals per day or less [AOR= 2.8, 95% CI (1.382, 5.794)] were 2.8 times more likely to develop depression as compared to those who eat more than two meals a day. Patients who had a history of ART adverse drug side effect [AOR = 4.7, 95% CI (1.317, 16.514)] were 4.7 times more likely to be depressed as compared to those who had not. The probability of occurring depression was 2.8 times higher among patients who were on WHO HIV/AIDS clinical stage II or above as compared to those on stage I [AOR= 2.8, 95% CI (0.142, 0.786)]. Depression was 2.4 times more likely to occur among those who live alone [AOR=2.4, 95 % CI (1.097, 5.429)] as compared to those who live with their parents (Table 3).

## 4. Discussion

In this study, the prevalence of depression among adult HIV/AIDS patients on ART was 14.6%, with 95% CI (10.9% - 18.2%), which is in line with a study conducted in Dilla, Ethiopia (11.2%) [19]. But it is relatively low as compared to 45.8% in Harar [14], 38.94% in Debrebirhan Referral Hospital [20], 43.9% in Tigray [21], 41.2% in Alert Hospital Addis Ababa [7], 63.1% in Khartoum, Sudan [22], and 26.7% in Cameroon [23]. The reason for this discrepancy might be attributable to several factors, including the population being studied, the study periods, the depression diagnostic tools difference, and the sample size used.

Nonadherence to ART was reported as a determinant factor for depressive symptoms, supported with studies done in Ethiopia [7], South Africa [24], and Cameroon [23] and studies from a review and meta-analysis in Korea [25]. In Ethiopia, depression was associated with less than 95% self-reported adherence [1]. An increased risk of nonadherence correlated with the higher prevalence of depression symptoms [26]. This consistently strong evidence implies that nonadherence to ART leads adult HIV/AIDS patients on ART to be depressed. This study confirms previous results on the influence of nonadherence to mental health.

Patients who eat two meals per day or less were more depressed as compared to those who eat more than two meals a day, consistent with a previous systematic review [27] which revealed that there was a positive relationship between frequent family meals and increased self-esteem. A study in Delhi, India, also reported that patients with nil or low self and family incomes had a greater prevalence of depression [12]. Though income did not show significant association with depression in this study, individuals who had enough income could have the probability to eat more than two meals a day. In turn, this self and family income might play an important role in the psychological and social stability of the patients.

Patients on WHO HIV/AIDS clinical stage II or above were more likely to develop depression as compared to that of patients with WHO HIV/AIDS clinical stage I. This finding was in line with the studies done in Addis Ababa, Ethiopia [7, 20]. A review and update on HIV and depression reported a significant association between depressive symptoms and HIV progression [28]. Another cohort study among pregnant women in Tanzania showed depressive symptoms among HIV-infected women were associated with a significantly increased risk of clinical disease progression to WHO HIV/AIDS clinical stages III and IV [29]. A review of longitudinal studies also reported that chronic depression is associated with clinical and immunological progression of HIV/AIDS [30]. Therefore, this significant association may be due to the chronic impact on immune and disease-related parameters, which in turn may develop depression symptoms, among adults on ART.

Depressions were more common among patients who had a history of ART adverse drug side effect as compared to those who had not. Although HIV/AIDS cannot be cured, medications can help keep people healthy. But since these medications have toxicities and adverse drug effects [31, 32], symptoms of depression could be related to medication side effects. Consistent with the previous studies, depression symptoms might be triggered by the side effects of ART medications. Medication side effects may interrupt the normal functioning of adult HIV/AIDS patients on ART, so that patients may feel hopeless and develop depression.

Patients who were living alone were more likely to have depression as compared to those living with their families. Feelings of exclusion or loneliness may lead to depression. This study corroborated to previous findings done in Cameroon [23], Delhi [12], Canada [33], and USA [34] where family and social support in an illness like HIV/AIDS is very important. It provides mental, economic, and social stability to the patients. It also provides warmth and care to the patient in need and decreases the stresses faced by the patient [12]. A study reported in Allegheny County, Pittsburgh, PA, the USA, in 2014 [34], living alone increases the risk for social isolation. Individuals who are socially isolated receive significantly less emotional and instrumental support than those who are not socially isolated, which in turn increases the risk for depression. Social support could play an important role in the relationship between living arrangements and depression.

This study was limited by the use of self-reported depression symptoms and adherence level of treatments. Therefore, participants could have over or under-reported level of depression symptoms and ART treatment adherence. The temporal problem may have occurred on the issue of whether depression causes HIV/AIDS progression or HIV/AIDS progression causes depression. However, the findings from this study were internally and externally valid and provide a clear picture of the situation on depression among adult HIV/AIDS patients attending ART clinics in Aksum town.

## 5. Conclusions

Though the prevalence of depression among adult patients with HIV/AIDS on ART was found relatively low as compared to other studies, it is still a mental health problem. Nonadherence to ART, eating two meals per day or less, having a history of ART adverse drug side effect, being on HIV/AIDS WHO clinical stage II or above, and living alone were found statistically significant determinants of depression among adult patients with HIV/AIDS attending ART clinic. Programs on counseling and close follow-up of adherence to ART, drug side effects, and nutrition should be strengthened. Health facilities should link adult patients with HIV/AIDS who live alone to governmental and nongovernmental social supporter organizations.

## Figures and Tables

**Table 1:** Sociodemographic and economic characteristics of adult HIV/AIDS patients on ART in Aksum town, Aksum, Ethiopia, 2015.

**Variable**	**Categories**	**Number (n)**	**Percent (**%**)**
**Sex**	Male	239	58.2
	Female	172	41.8
**Age**	18-29	77	18.7
	30-39	187	45.5
	40-49	109	26.5
	50-62	38	9.2
	Mean age (SD)	36.2 (9.2)	
**Religion**	Orthodox	362	88.1
	Muslim	47	11.4
	Catholic	2	0.5
**Ethnicity**	Tigray	410	99.8
	Amhara	1	0.2
**Residence**	Urban	367	89.3
	Rural	44	10.7
**Marital status**	Single	72	17.5
	Married	192	46.7
	Widowed	46	11.2
	Divorced	80	19.5
	Separated	21	5.1
**Educational level**	No formal education	86	20.9
	Primary education(1-8)	196	47.7
	Secondary education(9-12)	102	24.8
	College and above (>12)	27	6.6
**Occupation**	No occupation	63	15.3
	Government employed	77	18.7
	Business/self-employed	136	33.1
	Farmer	24	5.8
	Daily laborer	11	27.0
**Monthly income in ETB**	<=500	156	37.9
	501-750	50	12.2
	751-1000	104	25.3
	>=1001	101	24.6
**Food diversity**	Low	141	34.3
	Medium	243	59.1
	High	27	6.6
**Daily eating pattern**	Three meals or more		
	Two meals or less	239	58.2
		172	41.8

**Table 2:** Depression and clinical characteristics of adult HIV/AIDS patients on ART in Aksum town, Aksum, Ethiopia, 2015.

**Variable**	**Categories**	**Number (n)**	**Percent (**%**)**
**Depression status**	Depressed	60	14.6
	Not depressed	351	85.4
**ART Adherence status**	Adherent	274	66.7
	Nonadherent	137	33.3
**Duration of ART in months**	6-12	45	10.9
	13-24	52	12.7
	>=25	314	76.4
**Treatment level**	First line treatment	400	97.3
	Second line treatment	11	2.7
**ART Doses given per day**	Once a day	164	39.9
	Twice a day	234	56.9
	Three times a day	13	3.8
**Treatment other than ART**	Yes	81	19.7
	No	330	80.3
**Any side effect of ART medication**	Yes	29	7.1
	No	382	92.9
**Opportunistic infection**	Yes	33	8.0
	No	378	92.0
**Functional status**	Working	401	97.6
	Ambulatory	10	2.4
**WHO stage of HIV/AIDS**	Stage I	84	20.4
	Stage II and above	327	79.6
**CD4 count**	<=200	42	10.2
	201-500	161	39.2
	>=501	208	50.6
**Hemoglobin level**	Normal	345	83.9
	Low	66	16.1
**BMI**	Underweight	79	19.2
	Normal	321	78.1
	Overweight	11	2.7
**Living condition**	Lives alone	116	28.2
	Lives with parents	39	9.5
	Lives with family	256	62.3

**Table 3:** Determinant factors of depression among adult HIV/AIDS patients on ART in Aksum town, Aksum, Ethiopia, 2015.

**Explanatory variable**	**Categories**	**Depression**				**COR(95**%**CI)**	**AOR(95**%**CI)**
		**Yes**		**No**			
		**Number**	%	**Number**	%		
**Residence**	Urban	322	87.7	45	12.3	Ref	Ref
	Rural	29	65.9	15	34.1	3.7(1.843,7.431)	2.1(0.677, 6.457)
**Daily eating pattern**	Three meals or more	220	92.1	19	7.9	Ref	Ref
	Two meals or less	131	76.2	41	23.8	3.6(2.018,6.507)	2.8(1.382, 5.794)*∗*
**Food diversity**	Low	109	77.3	32	22.7	3.263(0.92,11.63)	1.1(0.235, 5.020)
	Medium	218	89.7	25	10.3	0.83(0.234,2.940)	0.6(0.138, 2.341)
	High	24	88.9	3	11.1	Ref	Ref
**Treatment other than**	Yes	57	70.4	24	29.6	0.29(0.161,0.524)	1.3(0.522, 3.074)
**ART**	No	294	89.1	36	10.9	Ref	Ref
**Duration on ART in**	6-12	38	84.4	7	15.6	1.2(0.500,2.845)	1.30(.440, 4.037)
**month**	13-24	41	78.8	11	21.2	1.74(0.828,3.644)	0.8(0.290, 2.498)
	>=25	272	86.6	42	13.4	Ref	Ref
**ART Adherence status**	Adherent	255	93.1	19	6.9	Ref	Ref
	Non-adherent	96	70.1	41	29.9	5.73(3.170,10.365)	3.3(1.436, 7.759)*∗*
**Side effect of ART**	Yes	8	27.6	21	72.4	23.1(9.585,55.609)	4.7(1.317,16.514)*∗*
**medication**	No	343	89.9	39	10.2	Ref	Ref
**Opportunistic infection**	Yes	15	45.5	18	54.5	9.6(4.505,20.457)	2.1(0.559, 8.130)
	No	336	88.9	42	11.1	Ref	Ref
**WHO HIV/AIDS**	I	47	14.4	280	85.6	Ref	Ref
**clinical stage**	II or above	13	15.5	71	84.5	1.1(0.560,2.126)	2.8 (0.142, .786)*∗*
**CD4 count**	<=200	34	81.0	8	19.0	1.8(0.748,4.348)	1.2(0.390, 3.408)
	201-500	133	82.6	28	17.4	1.6(0.896,2.909)	0.5(0.288, 1.480)
	>=501	184	88.5	24	11.5	Ref	Ref
**Functional status**	Working	350	87.3	51	12.7	Ref	Ref
	Ambulatory	1	10.0	9	90.0	61.8(7.664,497.749)	0.1(0.004, 1.295)
**Substance use**	**Yes**	15	57.7	11	42.3	5.03(2.184,11.576)	0.2(0.702, 6.000)
	**No**	336	87.3	49	12.7	Ref	Ref
**Stigma**	Yes	18	66.7	9	33.3	3.26(1.392,7.659)	1.3(0.318, 5.343)
	No	333	86.7	51	13.3	Ref	Ref
**Living condition**	Lives alone	93	80.2	23	19.8	1.9(1.064,3.521)	2.4(1.097,5.429)*∗*
	Lives with parents	31	79.5	8	20.5	2.0(0.848,4.812)	1.0(0.361, 2.946)
	Lives with family	227	88.7	29	11.3	Ref	Ref

*∗*P value less than 0.05.

## Data Availability

The data used to support the findings of this study are available from the corresponding author upon request.

## Abbreviations

AIDS: Acquired immune deficiency syndrome
AOR: Adjusted Odds Ratio
ART: Antiretroviral Therapy
CI: Confidence Interval
HI: Health Institution
HIV: Human Immunodeficiency Virus
MI: Mental Illness
PHQ: Patient Health Questionnaire
PLWHA: People living with HIV
WHO: World Health Organization.
